# Geometric Deep Learning sub-network extraction for Maximum Clique Enumeration

**DOI:** 10.1371/journal.pone.0296185

**Published:** 2024-01-16

**Authors:** Vincenza Carchiolo, Marco Grassia, Michele Malgeri, Giuseppe Mangioni

**Affiliations:** Dipartimento Ingegneria Elettrica Elettronica Informatica Università di Catania, Catania, Italy; University of Parma, ITALY

## Abstract

The paper presents an algorithm to approach the problem of Maximum Clique Enumeration, a well known NP-hard problem that have several real world applications. The proposed solution, called *LGP-MCE*, exploits Geometric Deep Learning, a Machine Learning technique on graphs, to filter out nodes that do not belong to maximum cliques and then applies an exact algorithm to the pruned network. To assess the *LGP-MCE*, we conducted multiple experiments using a substantial dataset of real-world networks, varying in size, density, and other characteristics. We show that *LGP-MCE* is able to drastically reduce the running time, while retaining all the maximum cliques.

## Introduction

Graphs, also called networks, are ubiquitous structures that represent relationships between objects or entities in a variety of domains such as social, biological, technological and information. In general, networks can be used to model complex systems (hence the name “complex networks”) and to study their properties, such as the spread of diseases in a population, the exchange of goods in a trade network, or the diffusion of information in a social network. For instance, in a social network, each person can be represented as a node and the edges between nodes represent friendships or interactions between them; in molecules, nodes represent atoms, and edges represent chemical bonds between them; in a recommender system, nodes represent users or items, and edges represent preferences or co-occurrences between them. Due to the importance of networks in many real-world applications, there is a growing interest in developing efficient algorithms to solve problems on graphs, including trainable ones. In fact, networks have gained tremendous attention also in Machine Learning due to their ability to capture complex dependencies and interactions between data points allowing also to take into account topological properties. Graph-based machine learning models leverage the graph structure to learn representations of nodes or entire graphs. In particular, in the last years a new branch of Deep Learning, called Geometric Deep Learning (also known as Graph Representation Learning) has emerged [1]. It aims at learning representations of graphs, nodes and edges into a Euclidean space in order to apply classical Deep Learning algorithms, and approach a variety of tasks like classification, regression, clustering, link prediction, etc. The most popular algorithms in this field are Graph Neural Networks (GNNs) [2, 3], which are a generalization of Convolutional Neural Networks (CNNs) to operate on graphs. More in detail, they perform message-passing between nodes in the graph, where each node aggregates information from its neighbors (using a learnable function) and then updates its own representation. In this way, the graph structure, along with the input node (and/or edge) features, is taken into account during the learning process. Some examples of Graph Neural Network layers include:

- Graph convolutional networks (GCNs) that are a special type of graph neural networks (GNNs) that use convolutional aggregations. Applications of the classic convolutional neural network (CNN) architectures in solving machine learning problems, especially computer vision problems, have been hugely successful
- GraphSAGE is a framework for inductive representation learning on large graphs, it that leverages node feature information (e.g., text attributes) to efficiently generate node embeddings for previously unseen data [4]
- Graph attention networks (GATs): it is a neural network architectures that operate on graph-structured data, leveraging masked self-attentional layers. They are able to attend over their neighborhoods’ features and enable specifying different weights to different nodes without requiring any kind of costly matrix operation [3]
- Graph Isomorphism Networks (GINs): GINs generalizes the Weisfeiler-Lehman graph isomorphism test and hence achieves higher discriminative power than other GNNs [5].

Unfortunately, many algorithms to analyze or manipulate graphs are computationally difficult (i.e., NP–hard) and cannot be employed in real-world applications due to the large size of the input data and the exponential time complexity of the algorithms. However, we often encounter specific instances of NP–hard problems that can be solved efficiently using ML techniques. By analyzing these specific instances, we can gain insights into the mechanism of the used ML technique and develop algorithms that work well in practice. Furthermore, understanding the properties of specific instances can also help us identify instances that are likely to be easy or hard to solve, which can be useful in designing approximation algorithms or heuristics. Therefore, analyzing specific problems can be a valuable approach to tackling NP-hard problems in practice.

In this paper, among the problems on networks that are NP–hard, we focus on the Maximum Clique Enumeration (MCE) problem. It is a fundamental graph problem that belongs to the class of combinatorial problems. In particular, it consists in enumerating all the cliques of maximum size in an undirected graph [6, 7]. Other than the pure theoretical aspects, finding the maximum cliques has several practical applications, for instance, Liuet Alii [8] use the MCE to understand the principles of cell organizations and discover protein complexes, Kose at Alii [9] derive structural information and correlations between different metabolite levels by enumerating all the maximum cliques. Other applications are presented in [10], where Fukagawa et alii proposed a method for transforming the Tree Edit Distance (TED) between unordered trees problem into a maximum vertex weight clique problem and Mori et al. [11] into a maximum clique problem. As mentioned above, the MCE problem is particularly challenging, especially for large networks. In the literature there are several heuristic solvers (see [12, 13]) that often provide suboptimal solutions in reasonable time, but only recently, the use of machine learning has been investigated [14–16]. For instance, in [17–20] the authors proposed a technique aiming at reducing the MCE search space by pruning the input networks, by removing those nodes that probably doesn’t belong to any maximum cliques. More precisely, each node *v* is removed if a classic machine learning algorithm predicts the probability it belongs to some maximum clique is lower than some threshold. Predictions are performed by using both network measures and statistical properties as features for each node in order to capture structural properties of the network. Here, we further enhance the state-of-the-art and propose a pre-processing step for the MCE problem inspired from those proposed in [17–19, 21]. We named our algorithm as Learning Graph Pruning for MCE (*LGP–MCE*). More specifically, we employ Geometric Deep Learning [22] to implement a network pruning strategy aiming at reducing the running time required to enumerate the maximum cliques. Exploiting the capabilities of Graph Neural Networks, we show that our method is able to drastically prune real-world networks by removing up to ∼99% of nodes and ∼99% of edges while preserving all the maximum cliques. Such pruning ratios translate into speed-ups up to 21K times for a variation of the Bron-Kerbosch algorithm [23] implemented in the *igraph* [24] network analysis software. We validate the proposed methodology by training and testing on already pruned instances. In particular, we first remove all the nodes not belonging to the *K*–core of the network, and then apply the same pipeline described above. While the vertex and edge pruning rates of *LGP-MCE* drop significantly on some of such *K*–core pruned networks, we note that they are way denser than the original ones, and thus harder to prune safely.

The paper is organized as in the following. Section *Materials and Methods* briefly introduces the MCE problem and related literature, discusses the proposed approach and lists the database used to validate the results. Finally, section *LGP-MCE* reports on results discussing them.

## Materials and methods

In this section we formalize the MCE problem and describe the heuristic used to reduce the time needed to solve the problems, finally we describe the experiments setup and provide reference to the used datasets.

### The Maximum Clique Enumeration problem

A clique in graph theory is a subset *C* of vertices in an undirected graph *G*(*V*, *E*) such that every pair of distinct vertices in *C* are adjacent, i.e., for every two vertices *u*, *v* ∈ *C* with *u* ≠ *v*, there exists an edge (*u*, *v*)∈*E* in the graph. In other words, a clique is a complete sub-graph of the original graph, meaning that all the vertices in *C* are pairwise connected. The size of a clique is the number of vertices it contains, and a maximum clique is a clique of the largest possible size in a given graph. The problem of finding a maximum clique in a graph is known as the maximum clique problem and is NP-hard.

The algorithms for finding and enumerating all the maximum cliques can be divided into the following three families:

1. **Exact** solvers utilize a branch and bound approach to find an optimal solution with exponential complexity. This method involves dividing the entire research space into a number of sub spaces called branches, and iteratively pruning branches that are not useful for the final solution. The decision of whether to remove a branch or not is called bounding operation. Overall, exact solvers remain a crucial tool in combinatorial optimization and continue to be an active area of research.
2. **Heuristic** solvers are algorithms that utilize probabilistic models to explore the graph’s subsets of vertices in the most efficient way possible, while not being certain about the solution’s accuracy. Unlike exact solvers, heuristic solvers require polynomial time to find a (often suboptimal) solution. Therefore, it is not guaranteed that a heuristic solver can find all the maximum size cliques of a graph, nor is it assured that the size of every clique found by the algorithm is accurate.
3. **Domain specific solver** solve the problem on graphs belonging to a specific domains taking advantage of the domain properties.

In the literature there are numerous algorithms that fall into the three families. For instance, the Bron-Kerbosch algorithm [25], used to determine all cliques of the maximum size of an undirected graph, is characterized by a computational complexity of *O*(3^(|*V*|/3)^), where |*V*| is the number of nodes of the graph. Xu and Zhang, in [26], propose an algorithm employs an upper bound on the maximum clique size and prunes the search space by eliminating vertices that are unlikely to be part of a maximum clique, and Tsubaki et Al., in [27], propose a method based on the observation that high-degree vertices are more likely to be part of maximum cliques, and thus, removing low-degree vertices can reduce the search space while maintaining the same maximum clique size. However, the last two approach do not explicitly reports on complexity but reports the results of several benchmarks that, they tell, outperform state-of-the-arts.

Fast Max-Clique Finder algorithm [28] is a heuristic solver whose computational complexity is *O*(|*V*|^2^), where it is the maximum degree within the graph. Cliquer is another very popular implementation of the algorithm defined by Östergård in 2002 [29], and adopts the branch and bound strategy. Since the performance of this algorithm depends on node sorting, it uses a heuristic approach based on vertex coloring. EmMCE [30] is an algorithm that uses external memory to store those networks that cannot be allocated within RAM memory due to excessive size. To improve running time, this algorithm also runs in parallel. Recently, Chatterjee and Al. [31] proposed two new heuristic algorithms for the maximum clique problem, based on local search and probabilistic pruning techniques. The algorithms are designed to balance exploration and exploitation of the search space and are shown to outperform existing state-of-the-art heuristic solvers on various benchmarks. For further discussion about the MCE algorithms, we refer the Reader to [32].

### *LGP*–*MCE*

Our heuristic-based approach draws inspiration from the work of Lauri et al. [17, 19, 20, 33]. It involves enhancing existing solvers by reducing the search space through node pruning in input networks. Specifically, we propose a pre-processing step where nodes are removed (pruned) based on a Machine Learning model’s predicted probability of not belonging to a maximum clique, as long as the probability falls below a defined threshold called “confidence threshold”. The confidence threshold allows for a trade-off between accuracy and pruning rates, enabling the *LGP-MCE* to be more flexible and tailored to specific application needs. In fact, higher thresholds may grant higher pruning rates and lower accuracy, while lower thresholds may grant lower pruning rates and higher accuracy. Importantly, *LGP-MCE* does not affect the computational complexity of solvers since the algorithms are not modified, but the proposed pre-processing step produces smaller input graphs, thereby improving overall computational time and memory usage.

*LGP-MCE* differs from previous approaches because it employs Graph Neural Network (GNN) layers instead of classic Machine Learning algorithms, like Multi-Layer Perceptrons and random forests, and it only performs a single pruning stage.

More in detail, we use a Geometric Deep Learning model with Graph Attention Network (GAT) [22, 34] layers stacked together with a Multi-Layer Perceptron that, given the node embeddings, performs regression on the nodes and returns a value ranging over [0, 1]. We choose the Graph Attention Network layers since they are particularly suitable for our task: they do not perform neighborhood sampling, and they leverage the attention mechanism, proposed in [35] for Natural Language Processing tasks, to assign a relative importance score (i.e., the *attention coefficient*) to each neighboring node. Furthermore, Graph Attention Network layers use self-attention (through self-loops) and allow multiple attention-heads to improve the prediction performance. As in [17], after the pre-processing stage, we feed the pruned network to an exact clique solver (specifically, to the one implemented in the Python *igraph* library) to retrieve all the maximum cliques. It’s important to emphasize that alternative clique-finding algorithms, including heuristic methods like MoMC [36], could be employed. The choice of different solvers might influence the overall computational time, yet it doesn’t impact our pre-processing step.

Our model is trained in a supervised manner on a set of real-world networks, where maximum cliques can be efficiently found, and then applied to larger networks. Each node’s training target is a binary value, depending on whether it belongs to a maximum clique or not, respectively. Regarding the input node features, we compute the normalized node degree, the Local Clustering Coefficient (LCC), the Chi-squared *χ*^2^ of normalized degree (with respect to neighboring nodes) and of the Local Clustering Coefficient, and the normalized *K*–core value. Such features are both local (i.e., they are computed on the neighborhood of each node) and global (i.e., they are computed on the whole network), and capture different aspects of the network topology. These features include a subset of those used in [17], since they can be computed in linear time with respect to the number of edges, and they are sufficient to achieve good results. Moreover, the local features are aggregated by the Graph Neural Network layers through a message passing algorithm with learned weights, producing higher-order node features that better capture the topology.

The computational complexity of the proposed pre-processing method is linear with the number of nodes and edges in the network. In fact, the most expensive node feature to compute is the *K*–core, which is:

$$\begin{array}{c} O(K\text{-core})=O(|V|+|E|) \end{array}$$ (1)

where |*V*| and |*E*| are the number of nodes and edges respectively. On the other hand, the GAT layers have the following computational complexity:

$$\begin{array}{c} O(\text{GAT})=h\cdot (|V|+|E|) \end{array}$$ (2)

where *h* is the number of attention heads used. Thus, the final computational complexity is:

$$\begin{array}{c} O(\text{LGP-MCE})=h\cdot (|V|+|E|) \end{array}$$ (3)

which is linear with respect to the number of nodes and edges. Thus, *LGP-MCE* is computationally efficient and introduces an overhead that is negligible with respect to the computational complexity of the exact clique solvers, and that can be applied to very large networks, as we will show in the experimental evaluation, where we use networks with up to 7 million edges. We also note that we train and validate the performance of the proposed approach on networks that have already been pre-processed by removing all the nodes that do not belong to the *K*–core, as detailed in the following.

### Experimental setup

In this Section we describe the experimental settings and the evaluation metrics used to assess the performance of *LGP-MCE*.

#### Dataset

We use real-world networks from various domains, including social, biological, and communication networks, obtained from NetworkRepository.com [37]. The networks are downloaded in batch and transformed into undirected, while self-loops and parallel edges are removed. Then, the networks are pruned by removing all the nodes that do not belong to the maximum *K*–core of the network. The final step, as elaborated in Section *LGP-MCE*, serves as a benchmark strategy utilized in other works. It is intended to extract the denser segment of the network. Although this process may result in the loss of some maximum cliques, the resultant network instance is notably denser than the original, serving as a hard to safely solve benchmark for evaluating the algorithm.

After such elaboration, we split the networks into training and test sets. In particular, for the training we use ∼350 networks with a number of nodes from 1K to 70K, a number of edges from 1K to 1.8M, and a maximum clique size from 5 to 108. For the test set, we use 32 networks with a number of nodes from ∼300K to ∼200K, a number of edges from 20K to 7M, and a maximum clique size from 15 to 200. The test networks are also various in terms of average degree, degree assortativity, density, clustering coefficient, and *K*–core value. For the full list and statistics of the test networks used in our experiments, see Table 1.

**Table 1:** Test networks used in our experiments, along with their statistics. The networks are already pruned using the largest *K*–core value, and are obtained from the Network Repository [37].

Network	|*V*|	|*E*|	〈*k*〉	Density	|MC|	#MC	*T*	〈LCC〉	Deg.	K-core
									Assort.	Num. max
BA-1-10-60-L5	804	46.4K	115.4	0.1438	61	4	0.254	0.811	-0.0557	60
BA-2-24-60-L2	10.7K	639.8K	119.7	0.0112	61	5	0.031	0.707	-0.0633	60
PLC-60-30-L2	117.5K	7.0M	119.8	0.0010	22	3	0.008	0.020	-0.0024	60
TSOPF-FS-b162-c4	10.6K	593.6K	112.3	0.0106	49	19.8K	0.031	0.746	-0.2587	73
af-shell1	170.3K	2.9M	33.7	0.0002	15	67.0K	0.601	0.603	0.4125	19
bio-pdb1HYS	6.0K	365.7K	121.4	0.0202	57	2	0.547	0.577	0.0266	74
bio-WormNet-v3	575	75.4K	262.2	0.4569	121	18	0.588	0.603	-0.0005	164
c-fat500-10	500	46.6K	186.5	0.3738	126	3	0.774	0.774	0.3297	185
c-fat500-5	500	23.2K	92.8	0.1859	64	3	0.771	0.771	0.5515	92
g7jac060	473	19.7K	83.2	0.1763	52	9	0.727	0.720	-0.0062	59
g7jac140	348	20.3K	116.8	0.3365	52	89	0.553	0.584	0.0279	67
geo1k-200k	623	112.5K	361.2	0.5807	150	10	0.723	0.754	-0.0017	252
heart1	2.2K	402.7K	367.5	0.1677	200	16	0.620	0.686	0.0857	275
heart2	1.6K	220.6K	280.7	0.1787	200	5	0.720	0.740	0.1485	207
nemeth22	9.5K	671.7K	141.9	0.0150	70	3.6K	0.731	0.745	0.0145	75
opt1	3.8K	251.0K	133.6	0.0356	60	158	0.509	0.577	0.1273	80
orani678	412	49.7K	241.2	0.5869	77	13.2K	0.435	0.397	-0.2262	192
pkustk03	56.3K	1.5M	51.7	0.0009	30	8	0.580	0.592	0.3029	35
pkustk06	14.5K	455.1K	62.7	0.0043	30	56	0.493	0.504	0.4469	47
pkustk08	15.4K	1.2M	152.2	0.0099	60	914	0.475	0.528	0.1250	86
pkustk13	78.9K	2.7M	69.4	0.0009	36	1.2K	0.499	0.644	0.0343	41
pkustk14	51.2K	3.6M	141.7	0.0028	60	2.6K	0.491	0.557	0.1125	80
ramage02	12.4K	1.1M	172.1	0.0139	68	640	0.479	0.535	0.1175	98
rma10	11.5K	443.2K	77.0	0.0067	44	444	0.606	0.663	0.1501	50
sc-pwtk	204.6K	5.3M	52.3	0.0003	24	31.7K	0.581	0.583	0.4679	35
socfb-Auburn71	1.0K	78.5K	155.2	0.1537	57	6	0.373	0.418	-0.0008	95
socfb-BU10	5.6K	256.8K	92.2	0.0166	38	4	0.171	0.201	0.0003	50
socfb-Colgate88	1.3K	72.4K	111.0	0.0851	33	6	0.269	0.296	-0.0009	65
socfb-Hamilton46	736	37.3K	101.4	0.1380	22	21	0.291	0.319	-0.0008	63
socfb-UF	2.3K	153.6K	136.4	0.0606	53	466	0.378	0.433	0.0000	83
t3dh	55.9K	1.5M	54.2	0.0010	20	11.6K	0.437	0.470	0.1078	29
tsyl201	20.5K	1.2M	117.7	0.0057	60	928	0.582	0.629	0.0262	83

The elaboration of the networks and the feature computation are performed using the graph-tool library [38], while the Maximum Cliques are found using the igraph library [24].

#### Model architecture

We use Geometric Deep Learning models with Graph Attention Network layers [22] to learn the node embeddings, which are then classified by a Multi-Layer Perceptron (MLP). The activation function used between the layers is the Exponential Linear Unit (ELU), whereas the output layer uses the Sigmoid function to produce values between 0 and 1. We build a model that also computes the graph embedding by aggregating all the node embeddings using the max-pooling operation. This embedding is then concatenated to the node embeddings and fed to the MLP, that aims at capturing the global topology of the network and improve the classification performance.

#### Training

We train our models in a supervised manner using an Adam optimizer [39] that is a computationally efficient algorithm that is able to deal with both sparse gradients and non-stationary pbjectives. Regarding the loss function, we use the Binary Cross Entropy, which is frequently employed in binary classification tasks in machine learning, as it quantifies the dissimilarity between predicted probabilities and actual binary labels [40]. Since the dataset is heavily unbalanced, we use different positive and negative weight in the loss function to account for the different number of positive and negative examples in the training set. The hyper-parameters are tested in a grid search, and the best performing model parameters are selected, during the training, based on the balanced accuracy and recall metrics on the training set itself. To make the training more efficient, we use the Early Stopping technique, which stops the training when the scores do not improve for a certain number of epochs.

#### Performance measures

To evaluate the performance of the pruning obtained with *LGP-MCE*, we define the following measures:

$$\begin{array}{cc} \text{similarity} & =\{\begin{array}{cc} 0 & {\text{if}\;|\text{MC}|}_{p}<\left|\text{MC}\right|\\ \frac{\#{\text{MC}}_{p}}{\#\text{MC}}\cdot 100 & \text{otherwise} \end{array}\\ \text{speedup} & =\frac{\text{min}\;\text{time}(G)}{\text{min}\;\text{time}(G_{p})}\\ N_{p}^{R} & =\frac{\left|V\right|-\left|V_{p}\right|}{\left|V\right|}\cdot 100\\ E_{p}^{R} & =\frac{\left|E\right|-\left|E_{p}\right|}{\left|E\right|}\cdot 100 \end{array}$$

where |MC|_*p*_, |MC|, #MC_*p*_, #MC are the size and number of maximum cliques respectively in the pruned (*G*_*p*_(*V*_*p*_, *E*_*p*_)) and original graph *G*(*V*, *E*). In particular, the similarity measures the percentage of cliques of maximum size held after the pruning stage, the speedup measures the improvement in computational time (i.e., the minimum time spent by the solver on the original graph over the minimum time spent on the pruned graph), and $N_{p}^{R}$ and $E_{p}^{R}$ are the percentage of removed nodes and edges (as a consequence of removing the nodes).

### Results

In this Section we present and discuss the results of the *LGP-MCE*, obtained using the Geometric Deep Learning models described in sub section *Model Architecture* and trained on the ∼350 networks described in Section *Dataset*. We note that, in order to align *LGP-MCE* with smarter pruning strategies and make the comparisons more meaningful, we prune all the networks (including the ones used for training) by computing the *K*–core and remove all the nodes that do not belong to the inner one (i.e., largest *k*). On the other hand, it makes the experimental data harder to prune further and, thus, to get higher speedups.

The models have a variable number of Graph Attention Layers (from 1 to 4) and a variable number of hidden units (from 5 to 40) and heads (from 5 to 15), while the Multi-Layer Perceptron used for classification is fixed to four layers with a decreasing number of units (from 100 to 1). The models are trained using the Adam optimizer with a learning rate of 0.003, a weight decay of 0.0001, a dropout rate of 0.3, and a batch size of 8 (where each network is a batch unit). We train the models for 200 epochs, select the model that provides the best balanced accuracy and ROC AUC on the training set, and stop the training if the such score does not improve for 50 epochs. We stress that testing multiple models is a highly parallelizable task, and thus, we can train and test multiple models in parallel in order to select the best one. Considering that the computational time may vary depending on the system load, we solve each instance 10 times and take the shortest time as the solver time. In the experiments, the confidence threshold *T* selections is crucial: a too low value may result in a significant increase in computational time since too little nodes are removed, while a too high value may result in a significant loss of cliques. Here, instead of fixing the threshold *T* a priori, we select it for each network in the test set starting with a very high threshold (e.g., *T* = 0.99), and decreasing it until a congruous number of cliques is found. While this strategy is not optimal, it is simple and effective. In fact, finding the exact maximum cliques is not feasible in most real applications due to the size of the networks and the exponential complexity of the problem, however a best effort approach is the only solution available. Moreover, very high threshold values translate in very high pruning rates, and thus in a significant reduction of the computational time. This makes testing multiple threshold values feasible, and allows us to select the threshold *T* for each network in the test set. As shown by Table 2, in which we report the 18 networks where the pre-processing step retains all the maximum cliques and provides speedups greater than 1.5 times, *LGP-MCE* obtains the optimal solution up to 21K times faster by removing up to 99.1% of the nodes and 99.40% of the edges. Networks that are harder to solve, like bio-WormNet-v3, also benefit from very high speedups (760*x*) and pruning rates (67.65% for the nodes and 79.98% for the edges), which also translate in a significant reduction of the memory usage.

**Table 2:** Node pruning results that provide an optimal solution on the real-world network instances already pruned using the *K*–core. For the speedup, we enumerate the cliques ten times on both the original and pruned instances, and take the ratio between the minimum CPU time. The node pruning rate is the ratio of nodes predicted to not be part of a maximum clique, while the edge pruning rate is the ratio of edges removed as a direct consequence of the removal of nodes. The solver time columns are the time taken by the exact clique solver before and after the pruning (in seconds), while the *s* column is the speedup.

Network	Pruning rate		Maximum clique		*s*
	Nodes	Edges	Size	Num.	
BA-1-10-60-L5	88.06%	91.56%	61	4	140.53
BA-2-24-60-L2	99.11%	99.40%	61	5	21935.18
PLC-60-30-L2	96.66%	98.85%	22	3	80.15
bio-WormNet-v3	67.65%	79.98%	121	18	760.72
bio-pdb1HYS	81.09%	91.09%	57	2	25.53
c-fat500-10	49.60%	57.71%	126	3	2.73
c-fat500-5	74.40%	78.20%	64	3	5.08
g7jac060	61.31%	72.12%	52	9	5.95
geo1k-200k	53.45%	68.51%	150	10	15.95
heart1	25.41%	36.01%	200	16	1.66
heart2	43.51%	55.07%	200	5	2.87
nemeth22	26.38%	37.24%	70	3625	2.13
orani678	21.36%	43.61%	77	13200	1.88
pkustk03	96.06%	97.24%	30	8	56.01
pkustk06	92.07%	93.64%	30	56	16.11
socfb-Auburn71	81.01%	90.83%	57	6	13.84
socfb-BU10	67.79%	80.36%	38	4	3.54
socfb-Colgate88	45.75%	58.66%	33	6	2.30

As said above, we should note that *LGP-MCE* may not guarantee the optimal solution, like the networks in Table 3, where the pre-processing step removes some maximum cliques. Yet, this is a false problem since the *LGP-MCE* still provides extreme speedups (up to 100*K* times faster) and pruning rates (up to 99.99% for the nodes and 99.99% for the edges), and thus, it is still very useful in practice since it is a very close approximation of best solution. Moreover, we stress that a possible strategy could be to tune the threshold *T* in order to find at least one maximum clique (or a clique of close size) in very short time, and then leverage this information to prune the original networks safely. In fact, since at least a clique with that size was already found, all the nodes with lower degree are guaranteed to not belong to any maximum clique, and can be safely removed.

**Table 3:** Node pruning results that keep at least one maximum clique on the real-world network instances already pruned using the *K*–core. For the speedup, we enumerate the cliques ten times on both the original and pruned instances, and take the ratio between the minimum CPU time. The node pruning rate is the ratio of nodes predicted to not be part of a maximum clique, while the edge pruning rate is the ratio of edges removed as a direct consequence of the removal of nodes. The solver time columns are the time taken by the exact clique solver before and after the pruning (in seconds), while the *s* column is the speedup. The % column is the similarity measure defined in subsection *Performance measures*, and quantifies the percentage of maximum cliques retained by the pre-processing step.

Network	Pruning rate		Maximum clique			*s*
	Nodes	Edges	Size	Num.	Simil.	
BA-1-10-60-L5	91.79%	95.41%	61	2	50.000%	439.40
BA-2-24-60-L2	99.41%	99.70%	61	1	20.000%	66872.56
PLC-60-30-L2	98.79%	99.52%	22	2	66.667%	180.05
TSOPF-FS-b162-c4	49.17%	66.27%	49	400	2.020%	7.31
af-shell1	99.97%	99.99%	15	1	0.001%	100197.95
bio-WormNet-v3	63.65%	77.72%	121	6	33.333%	1725.64
c-fat500-10	74.80%	83.11%	126	1	33.333%	8.86
c-fat500-5	87.20%	91.31%	64	1	33.333%	16.85
g7jac060	68.71%	78.55%	52	4	44.444%	7.94
g7jac140	67.82%	78.59%	52	11	12.360%	8.65
geo1k-200k	59.07%	74.54%	150	4	40.000%	35.94
heart1	85.49%	91.61%	200	1	6.250%	20.19
heart2	84.10%	90.42%	200	1	20.000%	15.28
nemeth22	98.32%	99.08%	70	24	0.662%	367.53
opt1	91.86%	96.21%	60	2	1.266%	44.52
orani678	22.82%	47.26%	77	3520	26.667%	3.96
pkustk03	99.77%	99.90%	30	1	12.500%	2062.88
pkustk06	96.53%	98.49%	30	16	28.571%	112.88
pkustk08	57.74%	71.30%	60	130	14.223%	5.31
pkustk13	98.00%	99.58%	36	2	0.163%	806.16
pkustk14	94.96%	99.10%	60	1	0.039%	369.98
ramage02	73.93%	80.55%	68	48	7.500%	5.75
rma10	97.45%	98.48%	44	1	0.225%	100.06
sc-pwtk	99.92%	99.98%	24	2	0.006%	17389.07
socfb-Hamilton46	65.08%	81.10%	22	2	9.524%	13.03
socfb-UF	39.12%	55.00%	53	8	1.717%	2.22
t3dh	91.86%	97.19%	20	24	0.206%	80.39
tsyl201	64.05%	82.45%	60	52	5.603%	9.12

## Conclusion

In this study, we introduce a Geometric Deep Learning-based node pruning pre-processing, that aims at reducing the computational time of Maximum Clique Enumeration solvers. We show that *LGP-MCE* is able to obtain extreme speed-up, while keeping all the maximum cliques. Furthermore, our experiments show that improved performance can be obtained by customizing the confidence threshold, specifically tailored to the characteristics of the network under analysis. We also emphasize that combining our pruning method with a heuristic solver, for example, might enhance solving times but could also elevate the risk of missing some maximum cliques. This configuration could prove beneficial in tackling exceedingly large instances that might otherwise be difficult to approach. These two aspects deserves further investigations that will be object of future works. Another direction for future works regards the extension of the method presented in this paper to find cliques in time varying networks [41].
